# Straightforward synthesis of a tetrasaccharide repeating unit corresponding to the *O*-antigen of *Escherichia coli* O16

**DOI:** 10.3762/bjoc.9.203

**Published:** 2013-08-28

**Authors:** Manas Jana, Anup Kumar Misra

**Affiliations:** 1 Bose Institute, Division of Molecular Medicine, P-1/12, C.I.T. Scheme VII-M, Kolkata-700054, India, Fax: 91-33-2355 3886

**Keywords:** *Escherichia coli*, glycosylation, lipopolysaccharide, *O*-antigen, tetrasaccharide

## Abstract

A straightforward synthesis of the tetrasaccharide repeating unit of the *O*-antigen of *Escherichia coli* O16 has been achieved following a sequential glycosylation strategy. A minimum number of steps was used for the synthesis of the target compound involving a one-pot glycosylation and a protecting group manipulation. All intermediate reactions afford their products in high yield, and the glycosylation steps are stereoselective.

## Introduction

**Figure 1:**
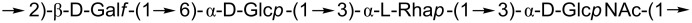
Structure of the tetrasaccharide repeating unit of the *O*-antigen of *Escherichia coli* O16.

Neonatal meningitis is a serious concern in developing countries [1]. The symptoms associated with this disease are unspecific and may ultimately lead to sepsis [2]. The common cause of the neonatal meningitis are bacterial infections in blood, and they start with the bacteria colonizing the gastrointestinal tract [3–4]. Microorganisms associated with neonatal meningitis are Streptococcus, *Escherichia coli* (*E. coli*) and *Listeria monocytogenes* [5–6]. Major *E. coli* strains causing neonatal meningitis are O1, O6, O7, O16, O18 and O83 [7]. Like many other *E. coli* strains, meningitis causing *E. coli* O16 is encapsulated and exhibits the K1 polysaccharides [8]. The structure of the *E. coli* O16 polysaccharide has been established by Jann et al. [9], which is a tetrasaccharide repeating unit containing D-glucosamine, L-rhamnose, D-glucose and D-galactofuranose moieties in a 1:1:1:1 ratio (Figure 1).

**Figure 2:**
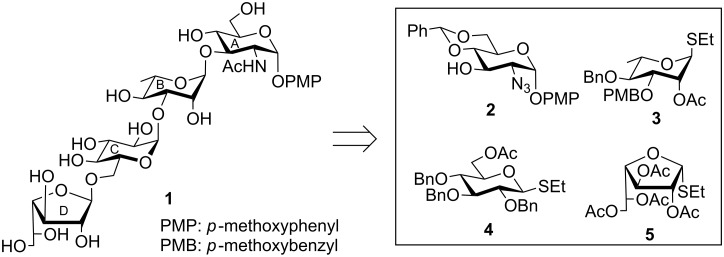
Structure of the synthesized pentasaccharide and its synthetic intermediates.

The emergence of multi drug resistant bacterial strains forces medicinal chemists to develop new approaches to combat bacterial infections. Since the structure of the *O*-antigen influences the virulence property of the pathogen, several reports appeared in the past on the development of glycoconjugate based therapeutics against bacterial infections [10–12]. Detailed biological studies of the glycoconjugates require a significant quantity of the oligosaccharides, which is difficult to isolate from natural sources. Hence, the development of chemical synthetic strategies for the synthesis of oligosaccharides is essential. In this context, a straightforward synthesis of the tetrasaccharide corresponding to the *O*-antigen of *E. coli* O16 as its *p*-methoxyphenyl glycoside has been developed and is presented herein (Figure 2).

## Results and Discussion

The target tetrasaccharide as its *p*-methoxyphenyl (PMP) glycoside was synthesized following a sequential glycosylation approach from the suitably functionalized monosaccharide intermediates **2** [13], **3** [14], **4** [15] and **5** [16]. These monosaccharide intermediates were prepared from the commercially available reducing sugars by using a number of recently developed reaction conditions (Figure 2). The notable features of the synthetic strategy include, (a) the use of thioglycosides as glycosyl donors in all glycosylation reactions; (b) the application of iodonium ion mediated glycosylation conditions; (c) the use of *p*-methoxybenzyl (PMB) ether protection as an in situ removable protecting group in a one-pot glycosylation reaction and its removal [17] and (d) the use of galactofuranosidic thioglycoside as a glycosyl donor.

**Scheme 1:**
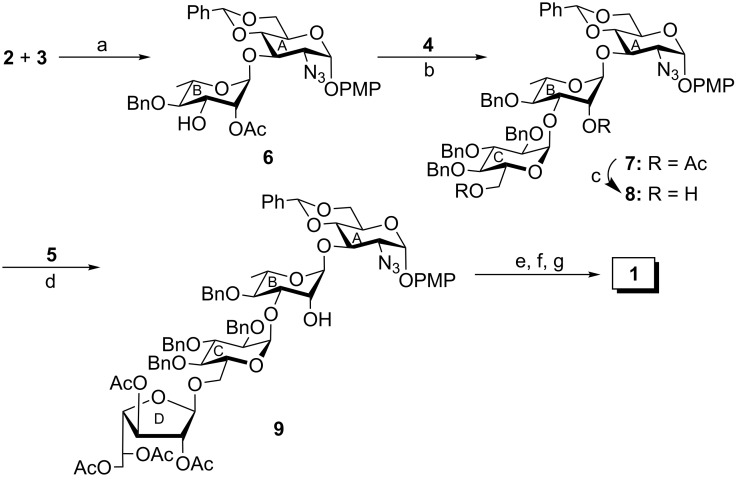
Reagents: (a) *N*-iodosuccinimide (NIS), TfOH, CH_2_Cl_2_, MS 4 Å, −30 °C, 1 h, then 0 °C, 1 h, 76%; (b) NIS, TfOH, CH_2_Cl_2_/Et_2_O (1:3, v/v), MS 4 Å, −40 °C, 1 h, 73%; (c) 0.1 M CH_3_ONa, CH_3_OH, room temperature, 2 h, 94%; (d) NIS, TfOH, CH_2_Cl_2_, MS 4 Å, −20 °C, 1 h, 72%; (e) Et_3_SiH, 10% Pd/C, CH_3_OH, AcOH, room temperature, 12 h; (f) acetic anhydride, pyridine, room temperature, 1 h; (g) 0.1 M CH_3_ONa, CH_3_OH, room temperature, 2 h, 64% in three steps.

The iodonium ion promoted stereoselective glycosylation of compounds **2** and **3** in the presence of a combination of *N*-iodosuccinimide (NIS) and triflic acid (TfOH) [18–19], followed by the removal of the *p*-methoxybenzyl (PMB) group in a one-pot reaction [17] by tuning the reaction conditions furnished the disaccharide acceptor **6** in 76% yield. The PMB group acted as an in situ temporary protecting group, which was removed after the glycosylation took place in the same pot. Formation of compound **6** was supported by its spectral analysis (signals at δ 5.46 (d, *J* = 3.0 Hz, H-1_A_), 5.09 (br s, 1H, H-1_B_) and at δ 98.1 (C-1_A_), 97.8 (C-1_B_) in the ^1^H and ^13^C NMR spectra respectively). The coupling of compound **6** with thioglycoside **4** in the presence of a combination of NIS and TfOH [18–19] in CH_2_Cl_2_/Et_2_O (1:3, v/v) furnished the 1,2-*cis* glycosylated compound **7** in 73% yield together with a minor quantity (~8%) of its other isomer, which was separated by chromatography. Spectral analysis of compound **7** confirmed its formation (signals at δ 5.46 (d, *J* = 3.5 Hz, H-1_A_), 5.11 (d, *J* = 3.5 Hz, H-1_C_), 5.06 (br s, H-1_B_) and at δ 98.1 (C-1_A_), 98.0 (C-1_B_), 92.5 (C-1_C_) in the ^1^H and ^13^C NMR spectra, respectively). The de-*O*-acetylation of compound **7** by using sodium methoxide furnished the trisaccharide acceptor **8** in 94% yield. The stereoselective glycosylation of compound **8** with D-galactofuranosyl thioglycoside **5** by using a combination of NIS/TfOH furnished the tetrasaccharide derivative **9** in 72% yield. The formation of compound **9** was supported by its spectral analysis (signals at δ 5.47 (d, *J* = 3.5 Hz, H-1_A_), 5.17 (br s, H-1_B_), 4.87 (d, *J* = 3.0 Hz, H-1_C_), 4.80 (br s, H-1_D_) and at δ 105.6 (C-1_D_), 99.9 (C-1_B_), 97.9 (C-1_A_), 93.8 (C-1_C_) in the ^1^H and ^13^C NMR spectra respectively). Compound **9** was subjected to a series of reactions involving (a) a catalytic transfer hydrogenation with triethylsilane and 10% Pd/C [20]; (b) an acetylation using acetic anhydride and pyridine, and (c) a saponification reaction with sodium methoxide to furnish compound **1**, which was purified over a Sephadex^®^ LH-20 gel to give the pure compound **1** in 64% overall yield. The structure of compound **1** was unambiguously confirmed by spectral analysis (signals at δ 4.89 (br s, H-1_D_), 4.88 (d, *J* = 3.0 Hz, H-1_A_), 4.84 (d, *J* = 3.0 Hz, H-1_C_), 4.76 (br s, H-1_B_) and at δ 107.6 (C-1_D_), 100.5 (C-1_B_), 95.9 (C-1_A_), 95.0 (C-1_C_) in the ^1^H and ^13^C NMR spectra respectively) (Scheme 1).

## Conclusion

In summary, a straightforward synthetic strategy was developed for the synthesis of the tetrasaccharide **1** as its *p*-methoxyphenyl glycoside corresponding to the *O*-antigen of *E. coli* O16. The target compound was synthesized by using a minimum number of steps and by applying recently developed elegant synthetic methodologies. Both the yields of the protecting group manipulations and the stereoselectivity of the glycosylation reactions were excellent.

## Experimental

General methods are similar as described in an earlier report [21].

***p*-Methoxyphenyl (2-*O*-acetyl-4-*O*-benzyl-α-L-rhamnopyranosyl)-(1→3)-2-azido-4,6-*O*-benzylidene-2-deoxy-α-D-glucopyranoside (6):** Similar as described in [21]. To a solution of compounds **2** (2 g, 5.00 mmol) and **3** (2.5 g, 5.42 mmol) in anhydrous CH_2_Cl_2_ (10 mL) was added MS 4 Å (2 g), and the reaction mixture was stirred under an argon atmosphere at room temperature for 30 min. The reaction mixture was cooled to −30 °C. To the cooled reaction mixture *N*-iodosuccinimide (NIS; 1.3 g, 5.77 mmol) and trifluoromethanesulfonic acid (TfOH; 50 μL) were added, and the mixture was stirred at the same temperature for 1 h. The temperature of the reaction mixture was raised to 0 °C and it was stirred at 0 °C for another 1 h. The reaction mixture was filtered through a bed of Celite^®^ and washed with CH_2_Cl_2_ (100 mL). The combined organic layers were successively washed with 5% Na_2_S_2_O_3_, satd. NaHCO_3_, and water, dried (Na_2_SO_4_), and concentrated. The crude product was purified over SiO_2_ by using hexane/EtOAc (4:1) as an eluant to give the pure compound **6** (2.6 g, 76%). Yellow oil; [α]_D_^25^ +77 (*c* 1.5, CHCl_3_); IR (neat): 3468, 2931, 2869, 2111, 1664, 1498, 1439, 1411, 1389, 1255, 1098, 1063, 1063, 1028, 661 cm^−1^; ^1^H NMR (CDCl_3_, 500 MHz) δ 7.45–7.23 (m, 10H, Ar-H), 7.01 (d, *J =* 9.0 Hz, 2H, Ar-H), 6.81 (d, *J =* 9.0 Hz, 2H, Ar-H), 5.50 (s, 1H, PhC*H*), 5.46 (d, *J* = 3.0 Hz, 1H, H-1_A_), 5.20–5.19 (m, 1H, H-2_B_), 5.09 (br s, 1H, H-1_B_), 4.72 (d, *J* = 11.5 Hz, 1H, PhC*H*_2_), 4.59 (d, *J* = 11.5 Hz, 1H, PhC*H*_2_), 4.30 (t, *J* = 9.5 Hz each, 1H, H-3_A_), 4.26–4.23 (m, 1H, H-6_aA_), 4.12–4.05 (m, 2H, H-3_B_, H-5_A_), 4.02–3.88 (m, 1H, H-5_B_), 3.78 (s, 3H, OC*H*_3_), 3.73–3.69 (m, 1H, H-6_bA_), 3.58 (t, *J* = 10.0 Hz each, 1H, H-4_A_), 3.42 (dd, *J* = 10.0, 3.5 Hz, 1H, H-2_A_), 3.26 (t, *J* = 9.5 Hz each, 1H, H-4_B_), 2.16 (s, 3H, COC*H*_3_), 0.86 (d, *J* = 6.0 Hz, 3H, CC*H*_3_); ^13^C NMR (CDCl_3_, 125 MHz) δ 169.9, (3 *C*OCH_3_), 155.6–114.7 (Ar-C), 102.1 (Ph*C*H), 98.1 (C-1_A_), 97.8 (C-1_B_), 81.5 (C-4_B_), 80.1 (C-4_A_), 74.8 (Ph*C*H_2_), 73.7 (C-3_A_), 72.7 (C-2_B_), 69.9 (C-3_B_), 68.7 (C-6_A_), 67.7 (C-5_B_), 64.1 (C-5_A_), 63.5 (C-2_A_), 55.5 (O*C*H_3_), 21.0 (CO*C*H_3_), 17.3 (C*C*H_3_); ESI–MS: 700.2 [M + Na]^+^; Anal. calcd for C_35_H_39_N_3_O_11_: C, 62.03; H, 5.80%; found: C, 61.86; H, 6.00%.

***p*-Methoxyphenyl (6-*O*-acetyl-2,3,4-tri-*O*-benzyl-α-D-glucopyranosyl)-(1→3)-(2-*O*-acetyl-4-*O*-benzyl-α-L-rhamnopyranosyl)-(1→3)-2-azido-4,6-*O*-benzylidene-2-deoxy-α-D-glucopyranoside (7):** Similar as described in [21]. To a solution of compounds **6** (2 g, 2.95 mmol) and **4** (1.7 g, 3.16 mmol) in anhydrous CH_2_Cl_2_/Et_2_O (10 mL; 1:3, v/v) was added MS 4 Å (2 g), and the reaction mixture was stirred under an argon atmosphere at room temperature for 30 min. The reaction mixture was cooled to −40 °C. To the cooled reaction mixture NIS (780 mg, 3.46 mmol) and TfOH (15 μL) were added, and the mixture was stirred at same temperature for 1 h. The reaction mixture was filtered through a bed of Celite^®^ and washed with CH_2_Cl_2_ (100 mL). The combined organic layers were successively washed with 5% Na_2_S_2_O_3_, satd. NaHCO_3_, and water, dried (Na_2_SO_4_), and concentrated. The crude product was purified over SiO_2_ by using hexane/EtOAc (3:1) as an eluant to give the pure compound **7** (2.5 g, 73%). Yellow oil; [α]_D_^25^ +67 (*c* 1.5, CHCl_3_); IR (neat): 3469, 2931, 2867, 2108, 1745, 1671, 1507, 1439, 1388, 1255, 1095, 1028, 865, 832, 753, 701, 660 cm^−1^; ^1^H NMR (CDCl_3_, 500 MHz) δ 7.43–7.09 (m, 25H, Ar-H), 7.01 (d, *J* = 9.0 Hz, 2H, Ar-H), 6.82 (d, *J* = 9.0 Hz, 2H, Ar-H), 5.53 (s, 1H, PhC*H*), 5.46 (d, *J* = 3.5 Hz, 1H, H-1_A_), 5.41–5.40 (m, 1H, H-2_B_), 5.11 (d, *J* = 3.5 Hz, 1H, H-1_C_), 5.06 (br s, 1H, H-1_B_), 5.03 (d, *J* = 11.0 Hz, 1H, PhC*H*_2_), 4.85 (d, *J* = 11.0 Hz, 1H, PhC*H*_2_), 4.84–4.80 (m, 2H, PhC*H*_2_), 4.68 (d, *J* = 12.0 Hz, 1H, PhC*H*_2_), 4.62 (d, *J* = 11.0 Hz, 1H, PhC*H*_2_), 4.52 (d, *J* = 10.5 Hz, 1H, PhC*H*_2_), 4.48 (d, *J* = 10.5 Hz, 1H, PhC*H*_2_), 4.26 (t, *J* = 9.5 Hz each, 1H, H-3_A_), 4.25–4.23 (m, 1H, H-6_aA_), 4.11 (dd, *J* = 10.0, 3.0 Hz, 1H, H-3_A_), 4.10–4.03 (m, 4H, H-5_A_, H-5_C_, H-6_aC_, H-6_bA_), 4.02–3.99 (m, 1H, H-5_B_), 3.93–3.90 (m, 1H, H-6_bC_), 3.77 (s, 3H, OC*H*_3_), 3.74–3.70 (m, 1H, H-4_C_), 3.65 (t, *J* = 9.0 Hz each, 1H, H-4_A_), 3.54 (dd, *J* = 10.0, 3.5 Hz, 1H, H-2_C_), 3.51 (t, *J* = 9.0 Hz each, 1H, H-3_C_), 3.45 (t, *J* = 10.0 Hz each, 1H, H-4_B_), 3.42 (dd, *J* = 10.0, 3.0 Hz, 1H, H-2_A_), 1.95, 1.89 (2 s, 6H, 2COC*H*_3_), 0.90 (d, *J* = 6.0 Hz, 3H, CC*H*_3_); ^13^C NMR (CDCl_3_, 125 MHz) δ 170.4, 170.3 (2 *C*OCH_3_), 155.8–114.7 (Ar-C), 102.0 (Ph*C*H), 98.1 (C-1_A_), 98.0 (C-1_B_), 92.5 (C-1_C_), 82.0 (C-4_C_), 79.9 (C-4_A_), 79.4 (2 C, C-3_B_, C-4_B_), 76.7 (C-3_C_), 75.8 (Ph*C*H_2_), 75.6 (Ph*C*H_2_), 74.9 (Ph*C*H_2_), 73.9 (C-2_C_), 72.1 (C-3_A_), 68.7 (C-6_A_), 68.5 (C-5_B_), 68.4 (C-5_C_), 67.6 (C-2_B_), 64.0 (C-5_A_), 63.6 (C-2_A_), 62.4 (C-6_C_), 55.5 (O*C*H_3_), 20.8, 20.7 (2 CO*C*H_3_), 17.3 (C*C*H_3_); ESI–MS: 1174.4 [M + Na]^+^; Anal. calcd for C_64_H_69_N_3_O_17_: C, 66.71; H, 6.04%; found: C, 66.54; H, 6.20%.

***p*-Methoxyphenyl (2,3,4-tri-*O*-benzyl-α-D-glucopyranosyl)-(1→3)-(4-*O*-benzyl-α-L-rhamnopyranosyl)-(1→3)-2-azido-4,6-*O*-benzylidene-2-deoxy-α-D-glucopyranoside (8):** Similar as described in [21]. A solution of compound **7** (2 g, 1.73 mmol) in 0.1 M CH_3_ONa in CH_3_OH (20 mL) was stirred at room temperature for 2 h. The reaction mixture was neutralized with Dowex 50W-X8 (H^+^) resin, filtered and concentrated. The crude product was passed through a small pad of SiO_2_ by using hexane/EtOAc (1:1) as an eluant to give the pure compound **8** (1.8 g, 94%). Yellow oil; [α]_D_^25^ +57 (*c* 1.5, CHCl_3_); IR (neat): 3458, 3002, 2932, 2871, 2472, 2109, 1747, 1667, 1505, 1439, 1411, 1389, 1255, 1097, 1063, 865, 754, 700, 663 cm^−1^; ^1^H NMR (CDCl_3_, 500 MHz) δ 7.46–7.17 (m, 25H, Ar-H), 7.02 (d, *J* = 9.0 Hz, 2H, Ar-H), 6.84 (d, *J* = 9.0 Hz, 2H, Ar-H), 5.50 (s, 1H, PhC*H*), 5.47 (d, *J* = 3.5 Hz, 1H, H-1_A_), 5.18 (br s, 1H, H-1_B_), 4.94–4.85 (m, 3H, PhC*H*_2_), 4.83 (d, *J* = 3.5 Hz, 1H, H-1_C_), 4.80 (d, *J* = 11.5 Hz, 1H, PhC*H*_2_), 4.69–4.56 (m, 4H, PhC*H*_2_), 4.36 (t, *J* = 9.5 Hz each, 1H, H-3_A_), 4.27–4.22 (m, 1H, H-6_aA_), 4.17–3.96 (m, 5H, H-2_B_, H-5_A_, H-5_B_, H-6_bA_, H-6_aC_), 3.92 (dd, *J* = 10.0, 3.5 Hz, 1H, H-3_B_), 3.84–3.80 (m, 1H, H-6_bC_), 3.78 (s, 3H, OC*H*_3_), 3.69 (t, *J* = 10.0 Hz each, 1H, H-4_A_), 3.58 (t, *J* = 9.5 Hz each, 1H, H-3_C_), 3.51 (t, *J* = 9.5 Hz each, 1H, H-4_C_), 3.49 (dd, *J* = 10.0, 3.5 Hz, 1H, H-2_C_), 3.48–3.40 (m, 3H, H-2_A_, H-4_B_, H-5_C_), 0.85 (d, *J* = 6.0 Hz, 3H, CC*H*_3_); ^13^C NMR (CDCl_3_, 125 MHz) δ 155.6–114.7 (Ar-C), 102.2 (Ph*C*H), 99.8 (C-1_A_), 97.9 (C-1_B_), 94.0 (C-1_C_), 82.2 (C-4_C_), 80.2 (C-4_A_), 78.9 (2 C, C-3_B_, C-4_B_), 77.4 (C-3_C_), 76.7 (C-2_C_), 75.6 (Ph*C*H_2_), 75.2 (Ph*C*H_2_), 74.9 (Ph*C*H_2_), 74.3 (Ph*C*H_2_), 74.0 (C-3_A_), 71.5 (C-5_C_), 68.7 (C-5_B_), 67.5 (C-2_B_), 64.3 (C-5_A_), 63.6 (C-2_A_), 61.2 (C-6_C_), 55.5 (O*C*H_3_), 17.2 (C*C*H_3_); ESI–MS: 1132.4 [M + Na]^+^; Anal. calcd for C_62_H_67_N_3_O_16_: C, 67.07; H, 6.08%; found: C, 66.88; H, 6.30%.

***p*-Methoxyphenyl (2,3,5,6-tetra-*O*-acetyl-β-D-galactofuranosyl)-(1→6)-(2,3,4-tri-*O*-benzyl-α-D-glucopyranosyl)-(1→3)-(2-*O*-acetyl-4-*O*-benzyl-α-L-rhamnopyranosyl)-(1→3)-2-azido-4,6-*O*-benzylidene-2-deoxy-α-D-glucopyranoside (9):** Similar as described in [21]. To a solution of compounds **8** (1.5 g, 1.35 mmol) and **5** (580 mg, 1.48 mmol) in anhydrous CH_2_Cl_2_ (5 mL) was added MS 4 Å (1 g), and the reaction mixture was stirred under an argon atmosphere at room temperature for 30 min. The reaction mixture was cooled to −20 °C. To the cooled reaction mixture NIS (350 mg, 1.56 mmol) and TfOH (5 μL) were added, and the mixture was stirred at the same temperature for an additional hour. The reaction mixture was filtered through a bed of Celite^®^ and washed with CH_2_Cl_2_ (50 mL). The combined organic layers were successively washed with 5% Na_2_S_2_O_3_, satd. NaHCO_3_, and water, dried (Na_2_SO_4_), and concentrated. The crude product was purified over SiO_2_ by using hexane/EtOAc (5:1) as an eluant to give the pure compound **9** (1.4 g, 72%). Yellow oil; [α]_D_^25^ +28 (*c* 1.5, CHCl_3_); IR (neat): 3469, 2931, 2867, 2108, 1745, 1671, 1507, 1439, 1388, 1255, 1095, 1028, 865, 753, 701, 660 cm^−1^; ^1^H NMR (CDCl_3_, 500 MHz) δ 7.46–7.14 (m, 25H, Ar-H), 7.03 (d, *J* = 9.0 Hz, 2H, Ar-H), 6.84 (d, *J* = 9.0 Hz, 2H, Ar-H), 5.52 (s, 1H, PhC*H*), 5.47 (d, *J* = 3.5 Hz, 1H, H-1_A_), 5.32–5.28 (m, 1H, H-5_D_), 5.17 (br s, 1H, H-1_B_), 5.00 (d, *J* = 2.0 Hz, 1H, H-2_D_), 4.95–4.89 (m, 3H, PhC*H*_2_), 4.87 (d, *J* = 3.0 Hz, 1H, H-1_C_), 4.82 (d, *J* = 11.0 Hz, 1H, PhC*H*_2_), 4.80 (br s, 1H, H-1_D_), 4.68 (d, *J* = 11.5 Hz, 1H, PhC*H*_2_), 4.57 (d, *J* = 11.5 Hz, 1H, PhC*H*_2_), 4.55 (d, *J* = 11.5 Hz, 1H, PhC*H*_2_), 4.53 (d, *J* = 11.5 Hz, 1H, PhC*H*_2_), 4.35 (t, *J* = 9.5 Hz each, 1H, H-3_A_), 4.27–4.22 (m, 2H, H-3_D_, H-6_aC_), 4.20–4.17 (m, 1H, H-4_D_), 4.12–4.07 (m, 3H, H-5_A_, H-6_aA_, H-6_bC_), 4.06–4.05 (m, 1H, H-2_B_), 4.02 (t, *J* = 9.5 Hz each, 1H, H-3_C_), 4.00–3.98 (m, 1H, H-5_B_), 3.93 (dd, *J* = 10.0, 3.5 Hz, 1H, H-3_B_), 3.89–3.85 (m, 1H, H-5_C_), 3.78 (s, 3H, OC*H*_3_), 3.75–3.72 (m, 1H, H-6_bA_), 3.65 (t, *J* = 9.5 Hz each, 1H, H-4_C_), 3.59 (t, *J* = 9.5 Hz each, 1H, H-4_A_), 3.52–3.49 (m, 1H, H-6_aD_), 3.46 (dd, *J* = 10.0, 3.0 Hz, 1H, H-2_C_), 3.43 (dd, *J* = 10.0, 3.0 Hz, 1H, H-2_A_), 3.40 (t, *J* = 10.0 Hz each, 1H, H-4_B_), 3.32–3.28 (m, 1H, H-6_bD_), 2.10, 2.04, 2.03, 1.97 (4 s, 12H, 4 COC*H*_3_), 0.85 (d, *J* = 6.0 Hz, 3H, CC*H*_3_); ^13^C NMR (CDCl_3_, 125 MHz) δ 171.2, 170.8, 170.7, 170.6 (4 *C*OCH_3_), 155.8–114.7 (Ar-C), 105.6 (C-1_D_), 102.2 (Ph*C*H), 99.9 (C-1_B_), 97.9 (C-1_A_), 93.8 (C-1_C_), 82.2 (C-3_C_), 81.2 (C-2_D_), 80.2 (C-4_A_), 79.4 (C-4_D_), 79.0 (C-2_C_), 78.9 (C-4_B_), 77.4 (C-4_C_), 76.4 (C-3_B_), 76.1 (C-2_B_), 75.6 (Ph*C*H_2_), 75.2 (Ph*C*H_2_), 74.8 (Ph*C*H_2_), 74.2 (Ph*C*H_2_), 74.1 (C-3_A_), 70.1 (C-5_C_), 68.9 (C-5_D_), 68.7 (C-6_A_), 67.6 (C-3_D_), 67.4 (C-5_B_), 66.0 (C-6_D_), 64.2 (C-5_A_), 63.4 (C-2_A_), 62.8 (C-6_C_), 55.6 (O*C*H_3_), 20.8 (2 C), 20.7 (2 C) (4 CO*C*H_3_), 18.1 (C*C*H_3_); MALDI–MS: 1462.5 [M + Na]^+^; Anal. calcd for C_76_H_85_N_3_O_25_: C, 63.37; H, 5.95%; found: C, 63.20; H, 6.18%.

***p*-Methoxyphenyl (β-D-galactofuranosyl)-(1→6)-(α-D-glucopyranosyl)-(1→3)-(α-L-rhamnopyranosyl)-(1→3)-2-acetamido-2-deoxy-α-D-glucopyranoside (1):** Similar as described in [21]. To a solution of compound **9** (1 g, 0.69 mmol) in CH_3_OH/AcOH (10 mL, 20:1, v/v), were added 10% Pd/C (100 mg) and Et_3_SiH (2 mL, 12.5 mmol), and the reaction mixture was stirred at room temperature for 12 h. The reaction mixture was filtered through a bed of Celite^®^, washed with warm CH_3_OH, and concentrated under reduced pressure. A solution of the crude product in acetic anhydride/pyridine (2 mL, 1:1 v/v) was kept at room temperature for 1 h and concentrated under reduced pressure. A solution of the acetylated crude product in 0.1 M CH_3_ONa in CH_3_OH (5 mL) was stirred at room temperature for 2 h. The reaction mixture was neutralized with Dowex 50W-X8 (H^+^) resin, filtered, and concentrated. The crude product was passed through a Sephadex^®^ LH-20 column by using CH_3_OH/H_2_O (2:1) as an eluant to give the pure compound **1** (345 mg, 64%). Glass; [α]_D_^25^ −6 (*c* 1.5, H_2_O); IR (KBr): 3466, 2945, 1632, 1376, 1165, 1067, 697 cm^−1^; ^1^H NMR (D_2_O, 500 MHz) δ 7.01 (d, *J* = 9.0 Hz, 2H, Ar-H), 6.87 (d, *J* = 9.0 Hz, 2H, Ar-H), 4.89 (br s, 1H, H-1_D_), 4.88 (d, *J* = 3.0 Hz, 1H, H-1_A_), 4.84 (d, *J* = 3.0 Hz, 1H, H-1_C_), 4.76 (br s, 1H, H-1_B_), 4.20–3.98 (m, 2H, H-2_D_, H-4_D_), 3.95–3.89 (m, 3H, H-2_A_, H-3_D_, H-5_B_), 3.88–3.80 (m, 3H, H-3_B_, H-5_C_, H-6_aC_), 3.76–3.68 (m, 5H, H-3_C_, H-4_A_, H-5_A_, H-6_abD_), 3.65 (s, 3H, OC*H*_3_), 3.64–3.62 (m, 2H, H-4_C_, H-5_D_), 3.61–3.56 (m, 2H, H-6_aA_, H-6_bC_), 3.55–3.50 (m, 2H, H-3_A_, H-6_bA_), 3.48–3.39 (m, 3H, H-2_B_, H-2_C_, H-4_B_), 1.94 (s, 3H, COC*H*_3_), 1.12 (d, *J* = 6.0 Hz, 3H, CC*H*_3_); ^13^C NMR (D_2_O, 125 MHz) δ 174.0 (*C*OCH_3_), 155.4–115.0 (Ar-C), 107.6 (C-1_D_), 100.5 (C-1_B_), 95.9 (C-1_A_), 95.0 (C-1_C_), 82.6 (C-3_D_), 80.8 (C-4_D_), 76.3 (C-5_B_), 72.8 (C-3_C_), 72.1 (C-4_C_), 71.5 (2 C, C-2_D_, C-3_A_), 70.7 (C-3_B_), 70.5 (C-2_B_), 70.1 (C-4_B_), 69.2 (2 C, C-2_C_, C-4_A_), 68.8 (C-5_D_), 68.3 (C-5_C_), 67.6 (C-5_A_), 66.1 (C-6_C_), 62.7 (C-6_D_), 60.5 (C-6_A_), 55.0 (O*C*H_3_), 54.9 (C-2_A_), 21.8 (CO*C*H_3_), 16.5 (C*C*H_3_); ESI–MS: 804.2 [M + Na]^+^; Anal. calcd for C_31_H_47_N_3_O_20_: C, 47.63; H, 6.06%; found: C, 47.46; H, 6.22%.

## Supporting Information

File 1 1D and 2D NMR spectra of compounds **1** and **6**–**9**.
