# Supplementary material for: Straightforward synthesis of a tetrasaccharide repeating unit corresponding to the O-antigen of Escherichia coli O16
Source: Beilstein J Org Chem. 2013 Aug 28;9:1757–62. doi: 10.3762/bjoc.9.203 (PMC3778367; doi:10.3762/bjoc.9.203)
Supplement: File 1 — 1D and 2D NMR spectra of compounds 1 and 6–9. [file Beilstein_J_Org_Chem-09-1757-s001.pdf]

Supporting Information

for

Straightforward synthesis of a tetrasaccharide repeating unit corresponding to the *O*-antigen of *Escherichia coli* O16

Manas Jana and Anup Kumar Misra*

Address: Bose Institute, Division of Molecular Medicine, P-1/12, C.I.T. Scheme VII-M, Kolkata-700054, India, Fax: 91-33-2355 3886

Email: Anup Kumar Misra - akmisra69@gmail.com

* Corresponding author

1D and 2D NMR spectra of compounds 1 and 6–9

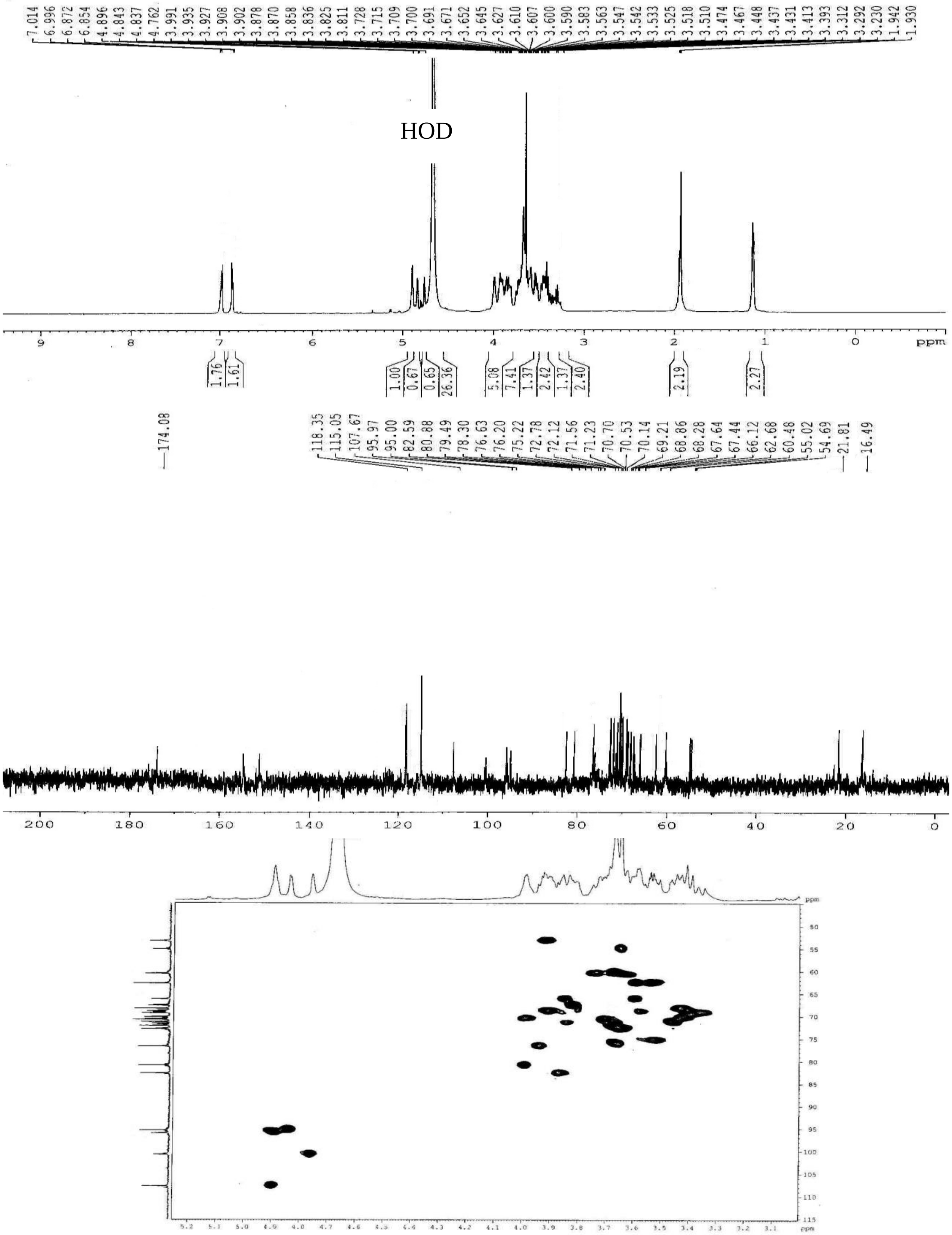

^{1}H, ^{13}C and 2D HSQC (selected region) NMR spectra of *p*-methoxyphenyl (β-D-galactofuranosyl)-(1→6)-(α-D-glucopyranosyl)-(1→3)-(α-L-rhamnopyranosyl)-(1→3)-2-acetamido-2-deoxy-α-D-glucopyranoside (**1**) (D_2O).

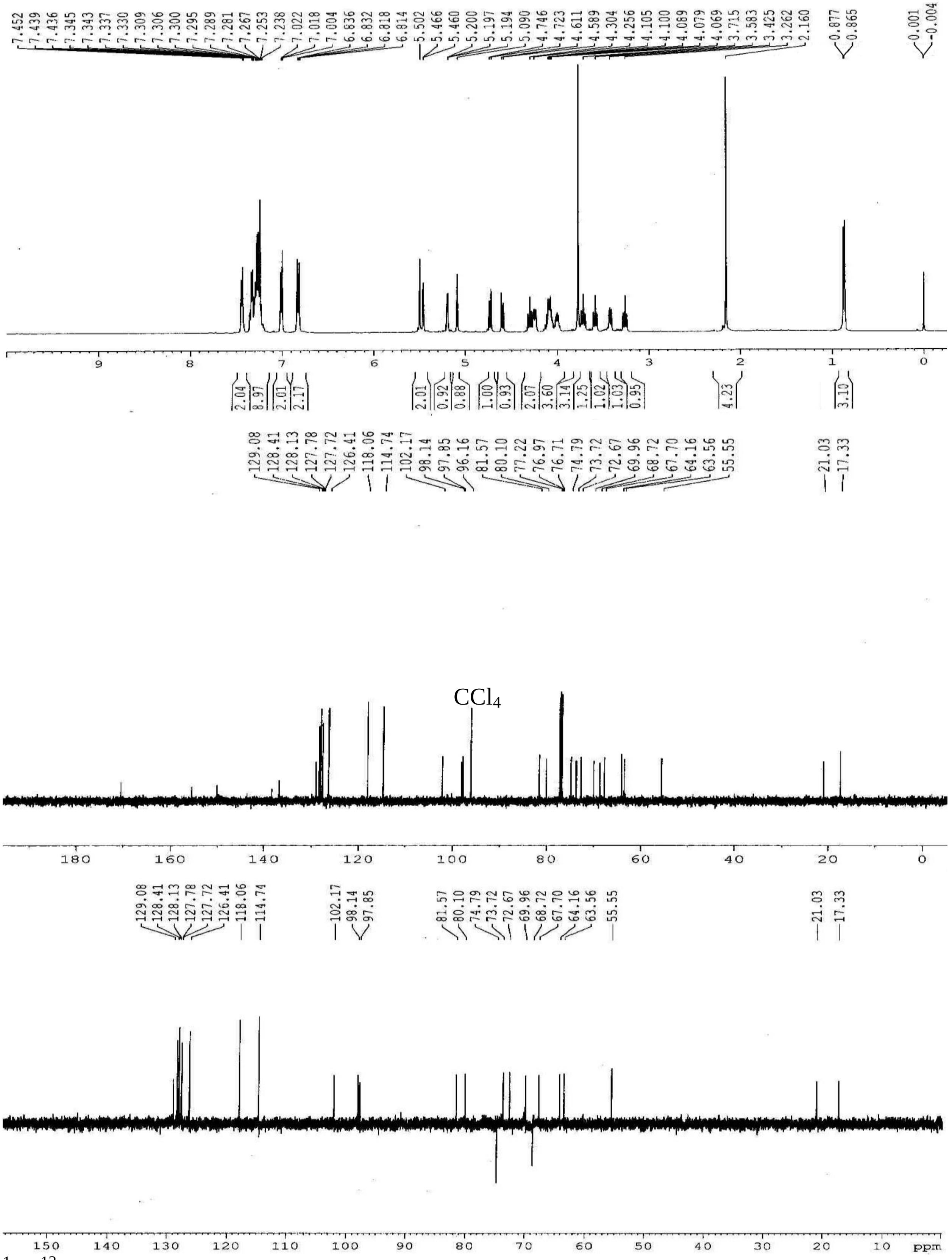

^{1}H, ^{13}C and DEPT 135 NMR spectra of *p*-methoxyphenyl (2-*O*-acetyl-4-*O*-benzyl-α-L-rhamnopyranosyl)-(1→3)-2-azido-4,6-*O*-benzylidene-2-deoxy-α-D-glucopyranoside (**6**) ($CDCl_3$/CCl_4).

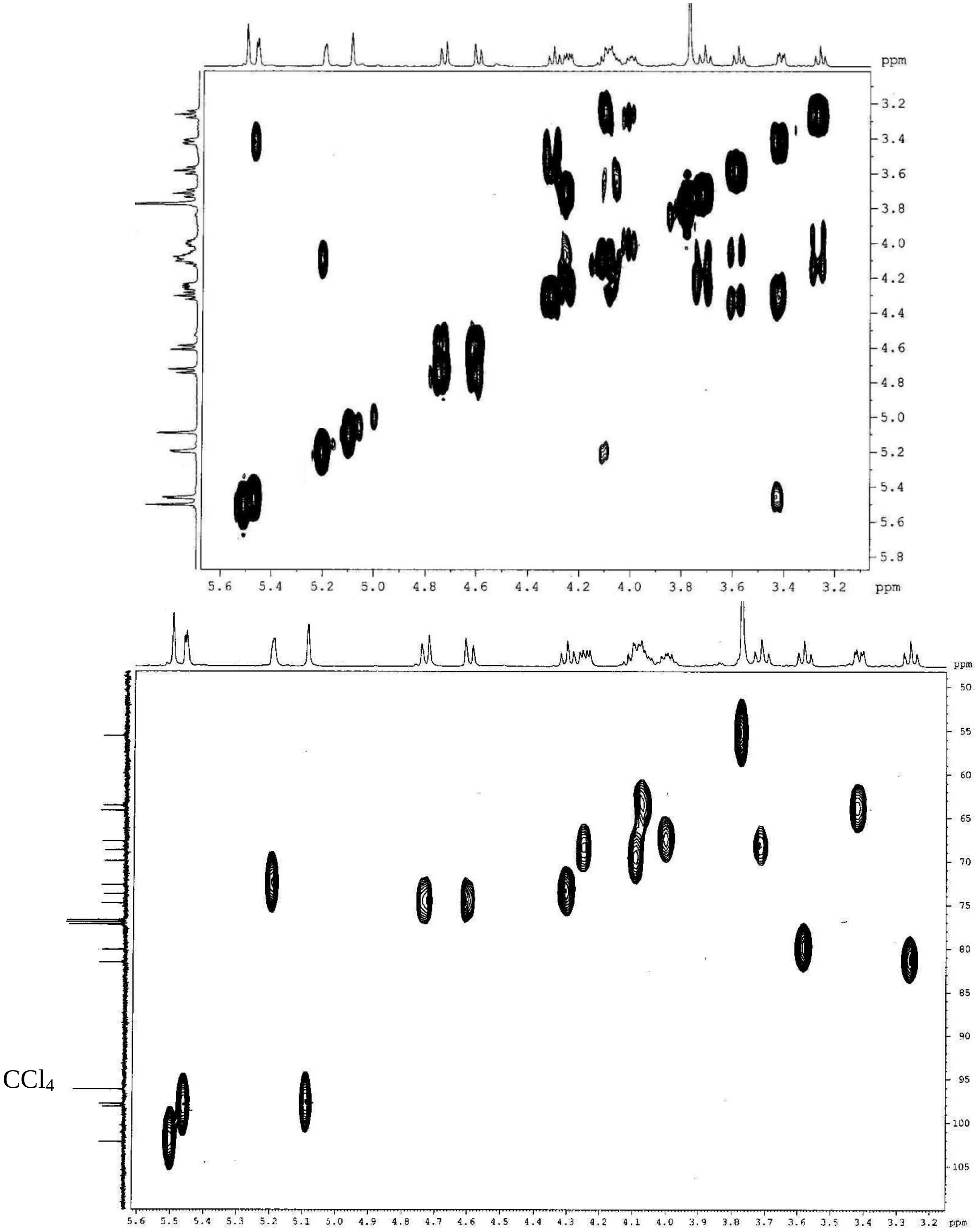

2D COSY and HSQC NMR spectra (selected region) of *p*-methoxyphenyl (2-*O*-acetyl-4-*O*-benzyl-α-L-rhamnopyranosyl)-(1→3)-2-azido-4,6-*O*-benzylidene-2-deoxy-α-D-glucopyranoside (**6**) ($CDCl_3/CCl_4$).

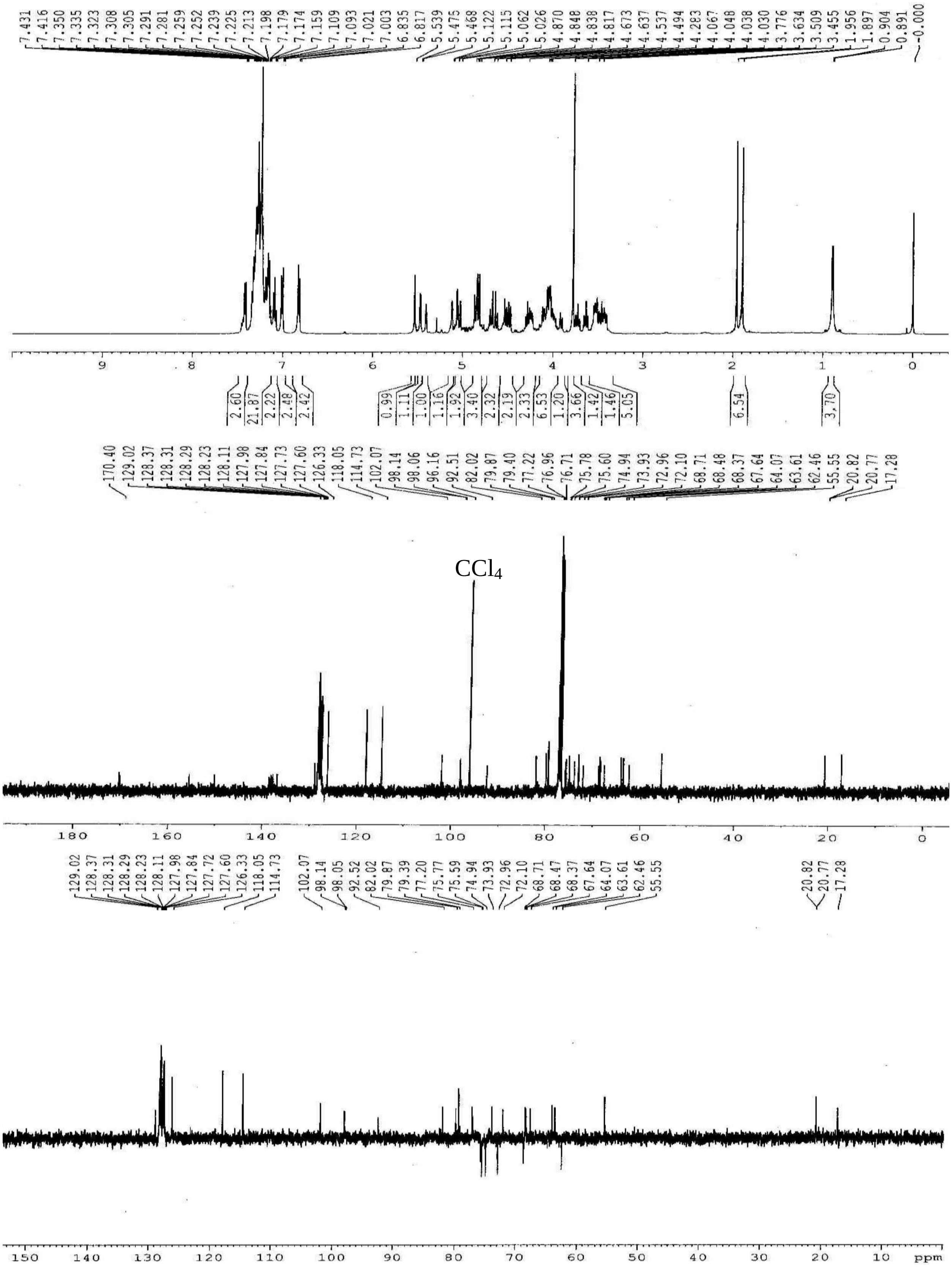

^{1}H, ^{13}C and DEPT 135 NMR spectra of *p*-methoxyphenyl (6-*O*-acetyl-2,3,4-tri-*O*-benzyl-α-D-glucopyranosyl)-(1→3)-(2-*O*-acetyl-4-*O*-benzyl-α-L-rhamnopyranosyl)-(1→3)-2-azido-4,6-*O*-benzylidene-2-deoxy-α-D-glucopyranoside (**7**) ($CDCl_3$/CCl_4).

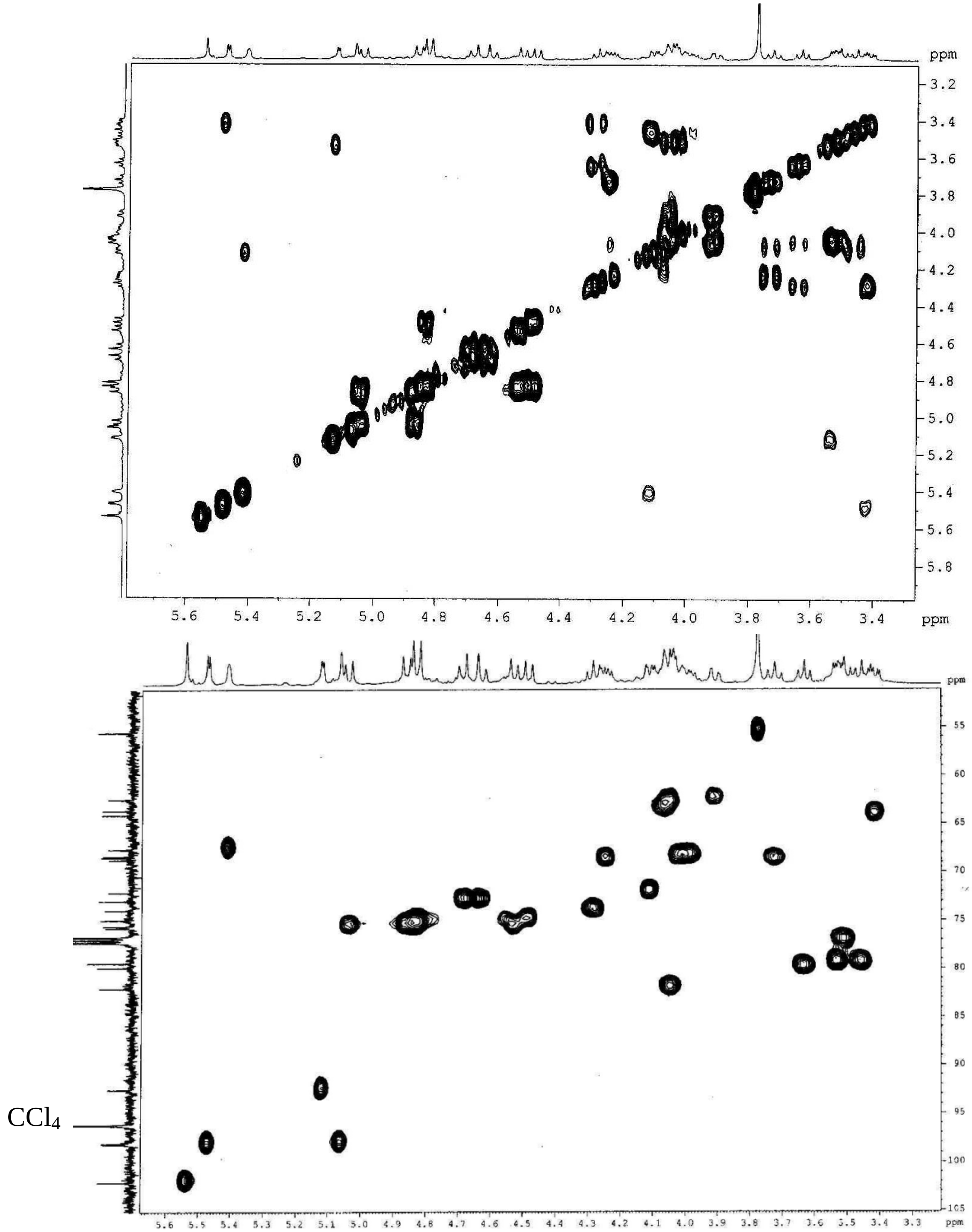

2D COSY and HSQC NMR spectra (selected region) of *p*-methoxyphenyl (6-*O*-acetyl-2,3,4-tri-*O*-benzyl-α-D-glucopyranosyl)-(1→3)-(2-*O*-acetyl-4-*O*-benzyl-α-L-rhamnopyranosyl)-(1→3)-2-azido-4,6-*O*-benzylidene-2-deoxy-α-D-glucopyranoside (**7**) ($CDCl_3$/CCl_4).

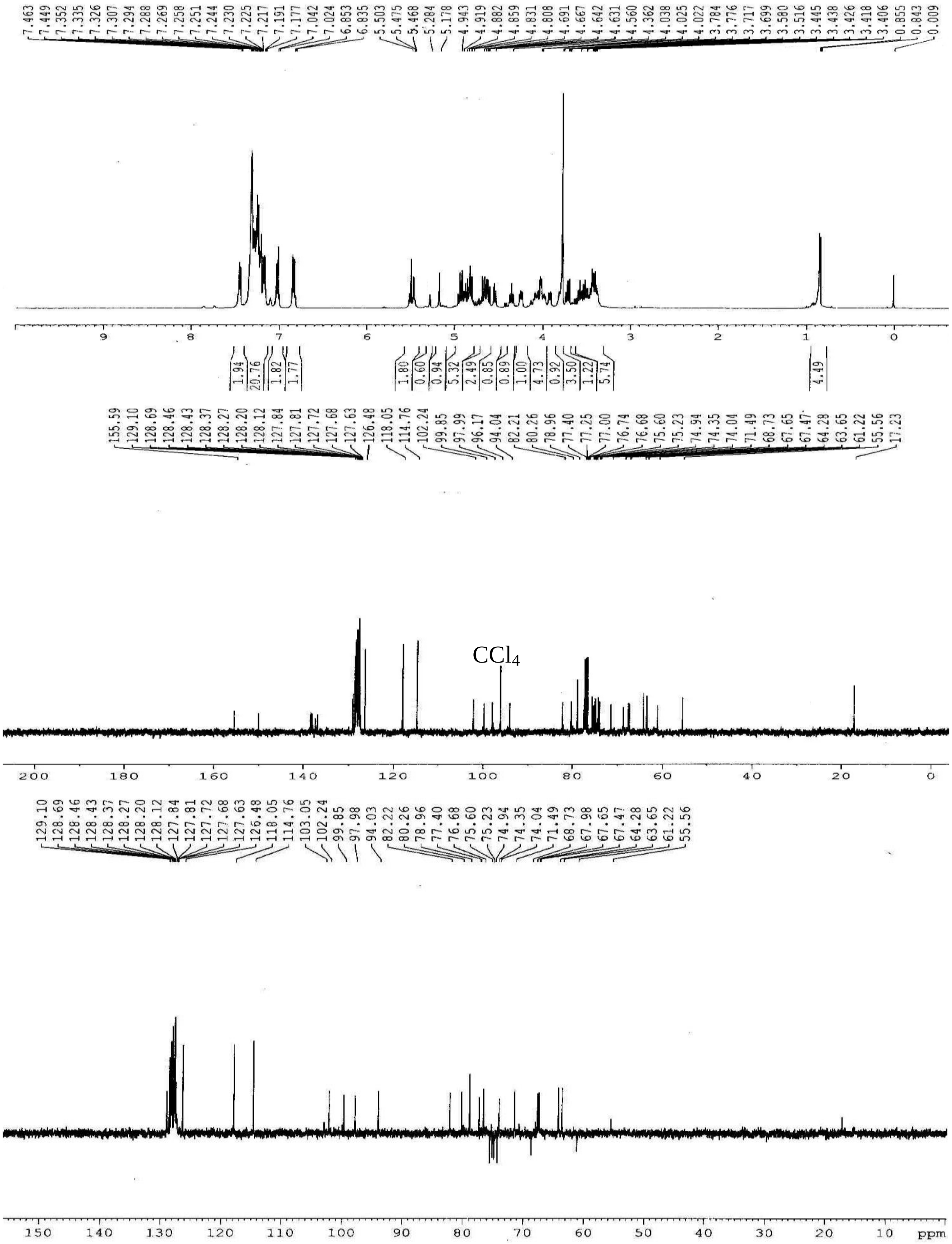

^{1}H, ^{13}C and DEPT 135 NMR spectra of *p*-methoxyphenyl (2,3,4-tri-*O*-benzyl-α-D-glucopyranosyl)-(1→3)-(2-*O*-acetyl-4-*O*-benzyl-α-L-rhamnopyranosyl)-(1→3)-2-azido-4,6-*O*-benzylidene-2-deoxy-α-D-glucopyranoside (**8**) ($CDCl_3$/CCl_4).

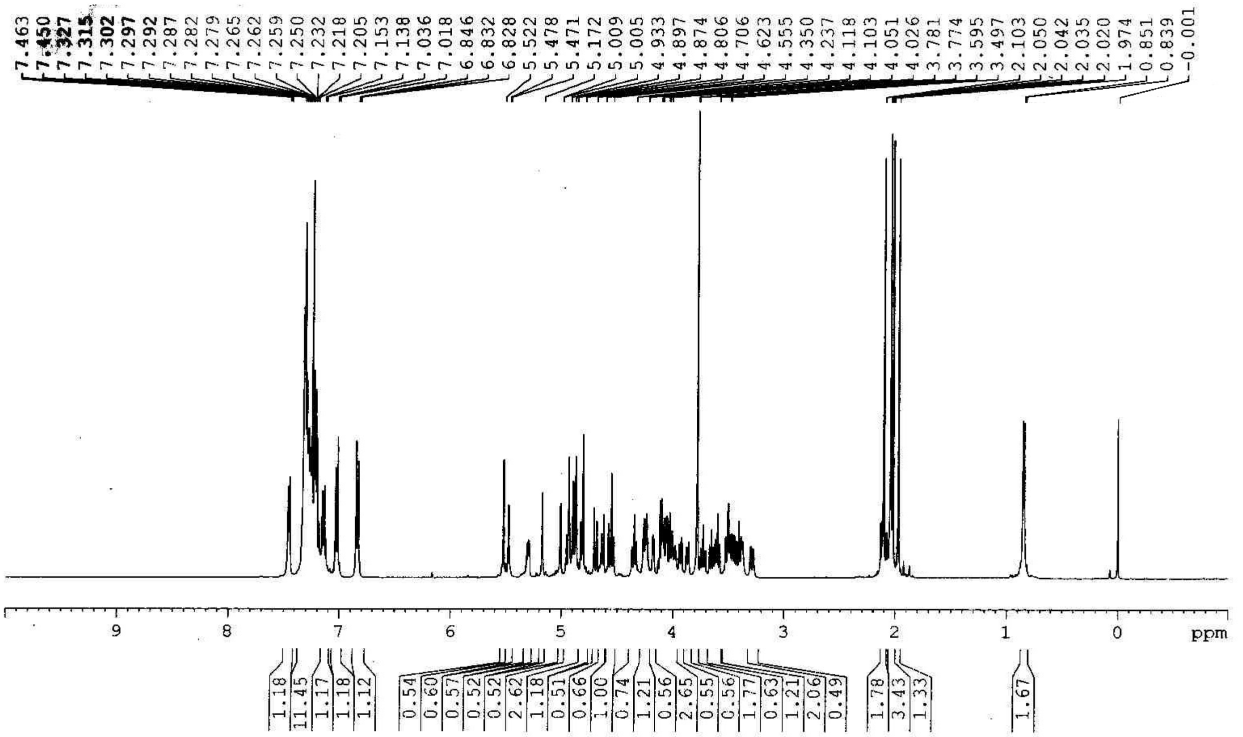

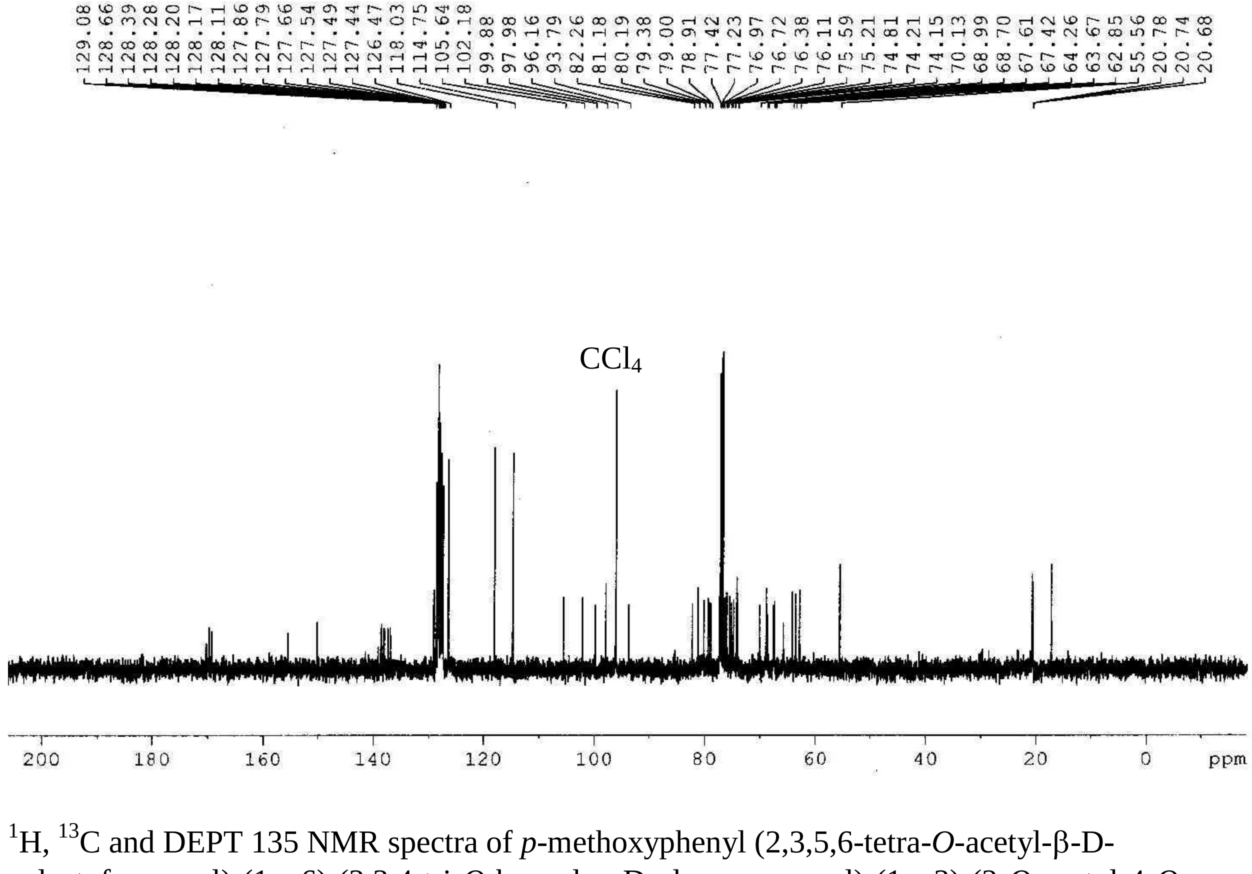

^{1}H, ^{13}C and DEPT 135 NMR spectra of *p*-methoxyphenyl (2,3,5,6-tetra-*O*-acetyl-β-D-galactofuranosyl)-(1→6)-(2,3,4-tri-*O*-benzyl-α-D-glucopyranosyl)-(1→3)-(2-*O*-acetyl-4-*O*-benzyl-α-L-rhamnopyranosyl)-(1→3)-2-azido-4,6-*O*-benzylidene-2-deoxy-α-D-glucopyranoside (**9**) ($CDCl_3$/CCl_4).

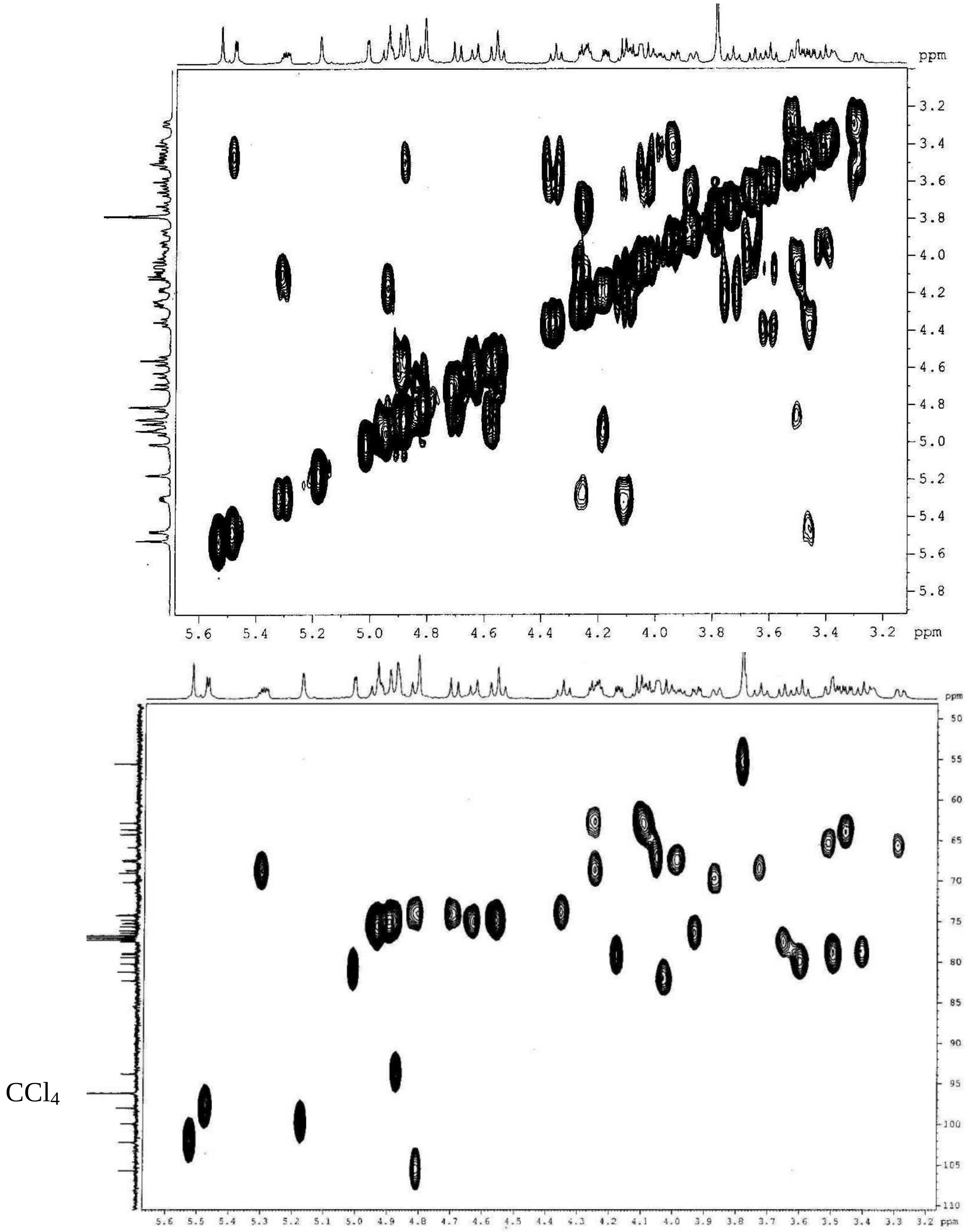

2D COSY and HSQC NMR spectra (selected region) of *p*-methoxyphenyl (2,3,5,6-tetra-*O*-acetyl-β-D-galactofuranosyl)-(1→6)-(2,3,4-tri-*O*-benzyl-α-D-glucopyranosyl)-(1→3)-(2-*O*-acetyl-4-*O*-benzyl-α-L-rhamnopyranosyl)-(1→3)-2-azido-4,6-*O*-benzylidene-2-deoxy-α-D-glucopyranoside (**9**) ($CDCl_3$/CCl_4).
